# SEX DIFFERENCES IN LINKS BETWEEN AGING EXPECTATIONS, PURPOSE IN LIFE, AND HEALTHY BEHAVIORS IN MIDLIFE KOREANS

**DOI:** 10.1093/geroni/igae098.3716

**Published:** 2024-12-31

**Authors:** Sun-ui Shin, Hyun-E Yeom, Eunyoung Park, Mi Sook Jung

**Affiliations:** Chungnam National University, Daejeon, Republic of Korea; Chungnam National University, Daejeon, Republic of Korea; Chungnam National University, Daejeon, Republic of Korea; Chungnam National University, Daejeon, Republic of Korea

## Abstract

Establishing a purpose in life and fostering healthy behaviors are critical for enhancing the quality of life in middle-aged populations. Negative perceptions of aging are closely linked to active self-care and overall well-being in aging individuals. This study aimed to investigate the mediating role of purpose in life for the relationship between aging expectations and healthy behaviors and to explore sex-related differences in these associations among midlife Koreans. A descriptive cross-sectional study was conducted with 298 community-dwelling adults aged 50 to 64 (mean age: 56.1 years; 154 males, 144 females). Data were collected through self-administered surveys and analyzed using Pearson’s correlation and PROCESS macro in SPSS version 26.0. Results indicated that aging expectations were significantly associated with purpose in life (r = -0.335, p < 0.001), and purpose in life was positively correlated with healthy behaviors (r = 0.276, p < 0.001). The influence of aging expectations on purpose in life differed by sex (β = -0.047, p = 0.020), with a more pronounced impact observed in males compared to females. Aging expectations indirectly affected healthy behaviors through purpose in life across both sexes, confirming the mediating role of life purpose. These findings suggest that aging expectations significantly influence the purpose in life, which subsequently leads to healthy behaviors in midlife adults, especially in men. The study underscores the importance of fostering positive perceptions of aging and a sense of purpose in life to support healthy aging, emphasizing the need to consider sex-related differences in these associations.

